# Obstructive Sleep Apnea: Emerging Treatments Targeting the Genioglossus Muscle

**DOI:** 10.3390/jcm8101754

**Published:** 2019-10-22

**Authors:** Olga Mediano, Sofia Romero-Peralta, Pilar Resano, Irene Cano-Pumarega, Manuel Sánchez-de-la-Torre, María Castillo-García, Ana Belén Martínez-Sánchez, Ana Ortigado, Francisco García-Río

**Affiliations:** 1 Sleep Unit, Pneumology Department, Hospital Universitario de Guadalajara, 19005 Guadalajara, Spain; sofiamp10@hotmail.com (S.R.-P.); presanob@gmail.com (P.R.); mariacastillogarcia37@gmail.com (M.C.-G.); 2 Medicine Department, Universidad de Alcalá, Alcalá de Henares, 28871 Madrid, Spain; 3 Centro de Investigación Biomédica en Red de Enfermedades Respiratorias (CIBERES), 28029 Madrid, Spain; sanchezdelatorre@gmail.com (M.S.-d.-l.-T.); fgr01m@gmail.com (F.G.-R.); 4 Pneumology Department, Hospital Universitario Ramón y Cajal, 28034 Madrid, Spain; irene.cano@yahoo.com; 5 Group of Translational Research in Respiratory Medicine, Hospital Universitari Arnau de Vilanova-Santa Maria, IRBLleida, 25198 Lleida, Spain; 6 Psychiatric Department. Hospital General La Mancha Centro, 13600 Toledo, Spain; martinez.anabelen@gmail.com; 7 Radiology Department, Hospital Universitario de Guadalajara, 19005 Guadalajara, Spain; anaorti_86@hotmail.com; 8 Pneumology Department, Hospital Universitario La Paz, IdiPAZ, 28046 Madrid, Spain

**Keywords:** Genioglossus muscle, sleep apnea, pharmacological treatment, hypoglossal nerve electrical stimulation, myofunctional therapy

## Abstract

Obstructive sleep apnea (OSA) is characterized by repetitive episodes of upper airway obstruction caused by a loss of upper airway dilator muscle tone during sleep and an inadequate compensatory response by these muscles in the context of an anatomically compromised airway. The genioglossus (GG) is the main upper airway dilator muscle. Currently, continuous positive airway pressure is the first-line treatment for OSA. Nevertheless, problems related to poor adherence have been described in some groups of patients. In recent years, new OSA treatment strategies have been developed to improve GG function. (A) Hypoglossal nerve electrical stimulation leads to significant improvements in objective (apnea-hypopnea index, or AHI) and subjective measurements of OSA severity, but its invasive nature limits its application. (B) A recently introduced combination of drugs administered orally before bedtime reduces AHI and improves the responsiveness of the GG. (C) Finally, myofunctional therapy also decreases AHI, and it might be considered in combination with other treatments. Our objective is to review these therapies in order to advance current understanding of the prospects for alternative OSA treatments.

## 1. Introduction

**Figure 1:**
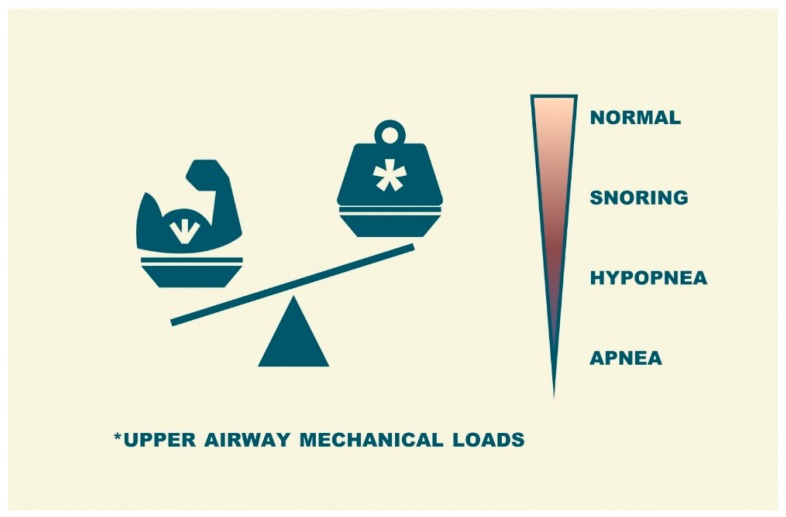
Representation of the imbalance produced during sleep between the loads on the upper airway and the function upper airway dilator muscles in obstructive sleep apnea (OSA) patients.

Obstructive sleep apnea (OSA) is a frequent condition characterized by a normal breathing pattern during wakefulness but repetitive episodes of upper airway obstruction during sleep [1]. These respiratory events (apneas or hypopneas) are due to a sleep-induced imbalance between the upper airway load (soft tissues and bony structures surrounding the upper airway can reduce the size of the resting pharynx) and upper airway dilator muscle tone (an alteration in dynamic neuromuscular response) (Figure 1) [2]. The severity of this disorder is usually expressed as the number of apnea/hypopnea events per hour of sleep time (apnea/hypopnea index or AHI).

In addition to its high prevalence [3], OSA has an important impact on quality of life [4] and increases the risk of accidents [5] as well as neurobehavioral and cardiovascular morbidity [6]. Therefore, the control of apneic events through appropriate treatment has a remarkable degree of clinical relevance and a large sociosanitary impact. Nevertheless, OSA is a heterogeneous and multifactorial condition whose treatment is not always easy and must be personalized for every patient [7].

Many OSA patients are obese, and the overloading of the upper airway during sleep is often a result of obesity because the increase in adipose tissue around the pharynx facilitates its collapse [8]. However, obesity is not the only risk factor for OSA. Tonsillar and adenoid hypertrophy, craniofacial abnormalities, and enlarged soft tissues around the pharynx are also common in these patients [2]. When reversible factors such as these are identified, the first-line therapy should be aimed at resolving these conditions (intensive lifestyle intervention, bariatric surgery, tonsillectomy, etc.). When etiological treatment is not possible or if, as usual, there is a multifactorial origin, continuous positive airway pressure (CPAP: positive pressure that keeps the upper airway open during sleep) is the gold-standard treatment for OSA patients [9]. Although CPAP is an effective treatment that can completely abolish respiratory events, it is associated with several difficulties. Above all, however, the main limitations of CPAP are its acceptance problems and a lack of continuous patient adherence in many cases, which may explain why randomized clinical trials failed to improve major health-threatening outcomes in non-sleepy subjects [10,11]. As an alternative, oral appliances (OAs: a device that enlarges the pharynx by moving the tongue and soft palate forward) are used to treat OSA in some subgroups of patients, mainly in non-obese patients with mild to moderate OSA or in severe OSA patients who are unable to use CPAP, but not as a first-line therapy for severe OSA. Although OAs are better tolerated, their efficacy is lower than that of CPAP and has considerable interindividual variability [12]. Clearly, OSA treatment is not uniform or simple. This issue is due to OSA’s multicomponent origin and the need to implement treatment in a personalized manner, with combined therapy as a possibility. Since there is no ideal treatment that applies to all patient types, improved knowledge of OSA pathophysiology will lead to important advances in its control. The previously mentioned treatment strategies focus on reducing collapsing upper airway forces; however, improving the dynamic neuromuscular response during sleep could also be effective for maintaining an open upper airway. Strategies aimed at improving the upper airway dilator muscle response might be useful for OSA treatment, at least in a certain group of patients.

Therefore, knowledge and understanding of the pathogenetic basis of OSA are important to implement new therapeutic strategies. As the genioglossus (GG) is the main dilator muscle of the upper airway, increasing the activity of this muscle and preventing hypotonia during sleep would be a promising therapeutic approach. The aim of this review is to describe and evaluate the evidence that supports new strategies targeted to improve GG dilator function in an attempt to increase upper airway patency in OSA patients.

## 2. Search Strategy and Study Selection

**Figure 2:**
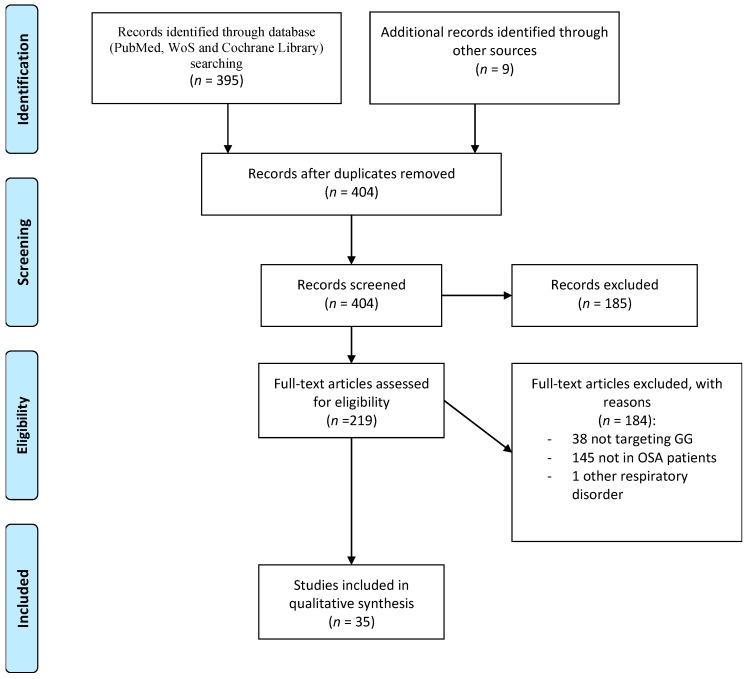
Schematic flow chart for the selection of studies.

To perform a systematic review on the most important aspects of this relationship, we analyzed the literature to detect all papers providing knowledge on the association in the PubMed, Web of Science, and Cochrane Library databases from inception to June 2019, focusing on the identification of reports about any interventions on the GG muscle in OSA patients. The studies were required to have a precise methodology while clearly presenting the importance and limitations of their results in the interpretation of the evaluated association. Studies were detected using the terms “sleep apnea” and “genioglossus muscle.” Three authors (O.M., S.R.-P., and P.R.) assessed the retrieved abstracts and full text of these studies to establish eligibility according to the inclusion criteria mentioned below. The inclusion criteria were as follows: (1) English articles published in peer-reviewed journals, (2) studies providing information targeted in the genioglossus muscle, and (3) studies of individuals with OSA. The exclusion criteria were as follows: (1) studies with fewer than 10 OSA patients; (2) editorials, case reports, and letters; and (3) studies conducted in children and adolescents (age <18 years). The first literature screening identified a total of 395 studies, and an additional nine articles previously identified by the authors were added. After the initial search of titles and abstracts, 185 articles were removed. A total of 219 full-text articles were assessed for eligibility, of which 184 were excluded: 38 studies did not include information about interventions targeting the GG, 145 studies did not include patients with OSA, and one study included patients with other respiratory disorders. Ultimately, 35 studies contained sufficient data to qualify for the present review and were included in our narrative synthesis (Figure 2). After locating and selecting these studies, we summarized the available evidence on electrical nerve stimulation, pharmacological treatment and myofunctional therapy. Finally, we considered their clinical applications.

There have been previous reviews in relation to emerging treatments for OSA, but this review adds to the field by focusing on a common strategy—increasing genioglossus muscle activity to improve OSA. Herein, we summarize these therapies and describe their clinical application. Furthermore, this review focuses on treatments that aim to improve the function of the genioglossus muscle after initial positive results from pharmacological treatments.

## 3. The Genioglossus Muscle

**Figure 3:**
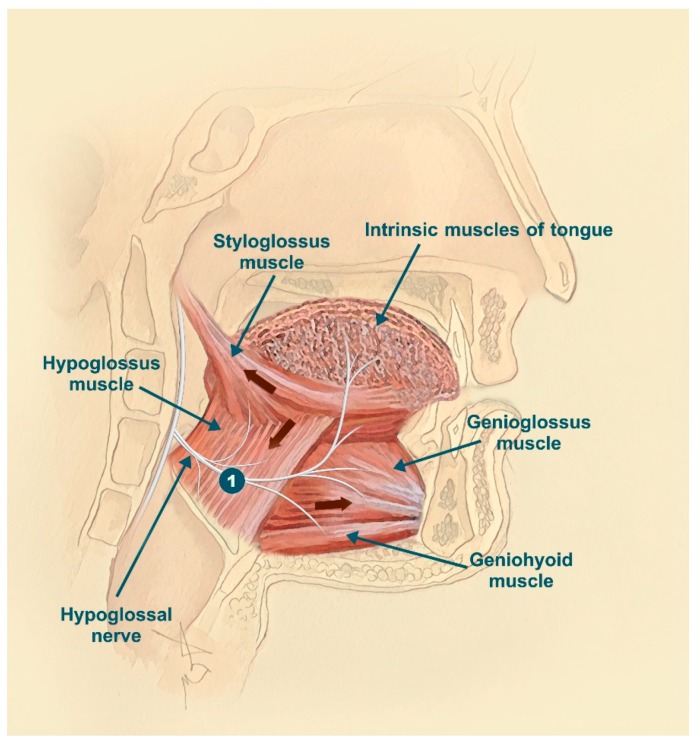
Hypoglossal nerve: course and branches. The genioglossus (GG) muscle is innervated by the medial branch of the hypoglossal nerve, increasing muscle activity during inspiration and reducing it during expiration. 1: correct position for electrical nerve stimulation.

The upper airway is a structure that consists of the nasal, pharyngeal, and laryngeal regions. It has a relevant role in three important human functions—breathing, swallowing, and speaking. The upper airway lacks rigid bony support to perform these important functions, with the surrounding muscles being responsible for its permeability; therefore, the upper airway is susceptible to collapsing forces. The upper airway is surrounded at least by 20 dilator muscles, and some are important for stabilizing and dilating it during sleep. The GG is one of the most extensively studied upper airway dilator muscles because of its accessibility and representativity, playing a very important role in upper airway patency during sleep [13]. From the mental symphysis of the mandible, the GG muscle enters the dorsum of the tongue; its main functions are tongue depression and protrusion [14]. The medial branch of the hypoglossal nerve innervates this muscle (Figure 3), decreasing muscle activity during expiration and increasing it during inspiration.

**Figure 4:**
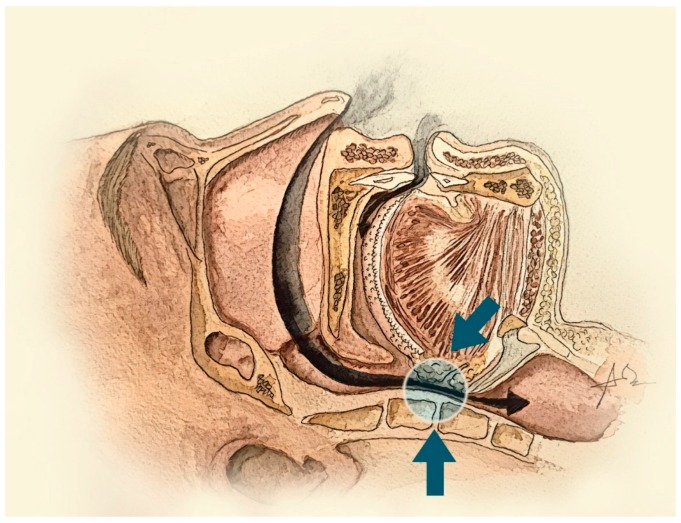
Representation of upper airway collapsibility in patients with OSA. The transition from wake to sleep decreases GG activity and increases upper airway resistance.

**Figure 5:**
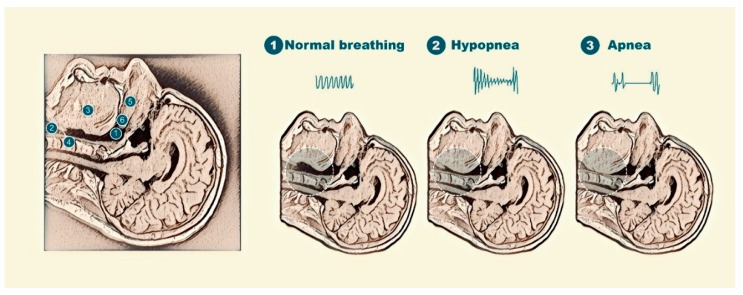
Image of the upper airway seen by magnetic resonance and its collapsibility in patients with OSA and anatomical description of the upper airway (left); representation of (1) normal breathing: 1: the pharynx; 2: the larynx; 3: the genioglossus muscle; 4: the epiglottis; 5: the hard palate; and 6: the soft palate; (2) partial upper airway obstruction; and (3) complete obstruction of the upper airway.

The three major inputs of GG activity are the sleep–wake state, a central pattern generator and chemo/mechanoreceptors. The most sleep state–related change that affects respiratory neural drive is the transition from wake to sleep, which decreases GG activity and increases upper airway resistance (Figure 4) [15]. The anatomical deficits existing in OSA patients are actively compensated by the upper airway dilator muscles during wakefulness. At the onset of sleep, GG activity falls farther and more quickly in OSA patients than in healthy subjects, producing upper airway obstruction (Figure 5) [13]. As the GG muscle is critical for maintaining upper airway patency during sleep and wakefulness, it has been proposed as a therapeutic target. Several therapeutic approaches are focused on different techniques to improve GG function in OSA patients.

## 4. Nerve Electrical Stimulation

Nerve electrical stimulation plays an increasingly significant role in OSA treatment, especially in those patients who do not tolerate CPAP. The target of electrical stimulation is the hypoglossal nerve (N. XII), the motor nerve that innervates the tongue muscles with the exception of the palatoglossus. Through stimulation of specific hypoglossal nerve fibers, the upper airway can be opened by protruding the tongue (Figure 3 represented by number 1).

In 1978, Remmers et al. [16] suggested a direct association between loss of GG muscle activity during sleep and upper airway collapsibility in OSA patients. Starting from this known pathophysiology, the hypothesis that OSA could be treated by neuromuscular electrical stimulation of the upper airway dilator muscles current to maintain upper airway patency during sleep has been developed [17]. Miki et al. [18] conducted initial human studies examining the tolerability and efficacy of upper airway stimulation (UAS); nevertheless, Schwartz et al. [19] did not report the result of stimulation of the hypoglossal nerve with respect to OSA in an animal model until 1993. Moreover, it was in 2001 that Schwartz et al. [20] described the implantation of a neurostimulator acting selectively on the fibers of the hypoglossal nerve that control tongue protrusion.

**Figure 6:**
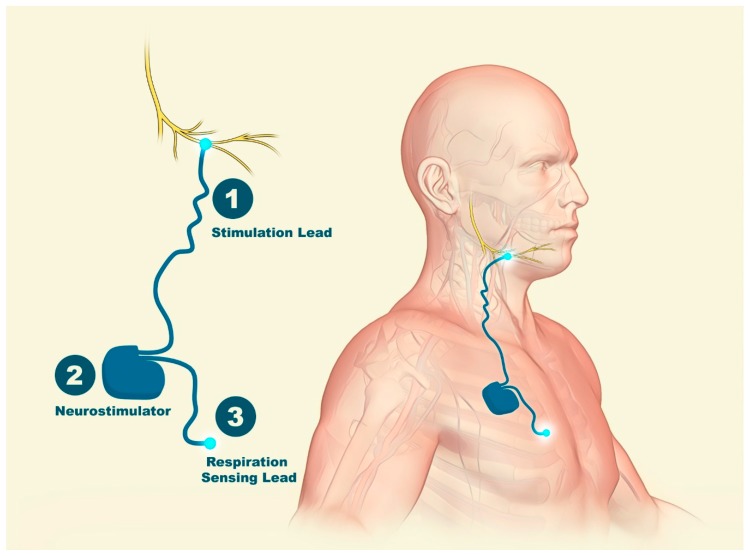
Hypoglossal nerve stimulation devices. (1) An electrode cuff wrapped around the hypoglossal nerve attached to (2) an implantable pulse generator (IPG) surgically placed in a subcutaneous pocket; the IPG is attached to a respiration-sensing lead (3).

**Figure 7:**
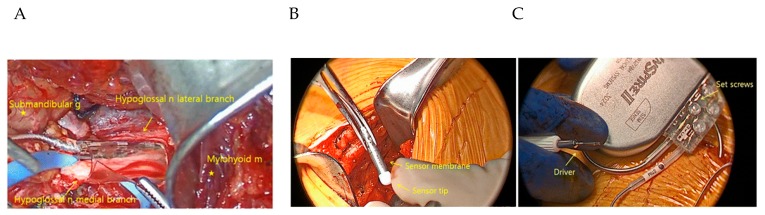
Hypoglossal nerve stimulation. (**A**) Cuff electrodes encircling the medial branch of the hypoglossal nerve (nerve = n, muscle = m, gland = g). (**B**) A pleural pressure-sensing lead is placed with the ventilatory sensor facing the pleura. (**C**) Implantable pulse generator (IPG) with profile connector ports that house the stimulation and pleural pressure-sensing lead connectors. From Hong et al. with permission [24].

Hypoglossal nerve stimulation devices typically comprise an implantable pulse generator (IPG) that is situated surgically in an infraclavicular subcutaneous pocket superficial to the pectoralis major muscle within the chest wall (Figure 6 and Figure 7). An electrode cuff attached to the IPG wraps around the distal portion of the hypoglossal nerve [13,21]. These hypoglossal nerve stimulation systems can incorporate an implantable chest sensor that monitors respiratory effort. The surgical procedure is performed under general anesthesia and started with the identification of the hypoglossal nerve [22]. An intimate understanding of N. XII anatomy is required, along with intraoperative neuromonitoring, to accurately place the stimulation electrode for selective UAS [23].

Different systems of nerve electrical stimulation therapy have evolved significantly over the past years, each offering different ways of acting: (1) Inspire I (Inspire Medical Systems^TM^, Maple Grove, MN, USA), an IPG with a tripolar half-cuff nerve stimulation electrode and a respiratory sensing lead placed against the pleura to detect respiratory effort [25], was reported to significantly improve AHI (52.0 ± 20.4 to 22.6 ± 12.1; *p* < 0.001) in patients with moderate to severe OSA [20], but initial technical difficulties such as sensor or hardware malfunction and broken electrodes occurred; (2) a system from Apnex Medical (Apnex Medical, Inc., St. Paul, MN, USA), was reported to significantly improve AHI values (43.1 ± 17.5 to 19.5 ± 16.7 events per hour, *p* < 0.05) and Epworth Sleepiness Scale (ESS) scores (12.1 ± 4.7 to 8.1 ± 4.4, *p* < 0.05) from baseline [26,27]; (3) the Aura 6000 Targeted Hypoglossal Neurostimulation (THN) system (ImThera Medical, Inc., San Diego, CA, USA) is characterized by continuous nerve stimulation without a respiratory pressure sensor, and the hypoglossal cuff electrode is placed more proximally than that of a typical IPG [28]. After 12 months of follow-up, the authors reported significant reductions in AHI (45 ± 18 to 21 ± 16.5 per hour, a 53% reduction; *p* < 0.001), 4% oxygen desaturation index (ODI, from 29 ± 20 to 15 ± 16 per hour; *p* < 0.001), and arousal index (AI) values (from 37 ± 13 to 25 ± 14 events per hour; *p* < 0.001) [28]. However, ESS scores did not improve (from 11 ± 7 to 8 ± 4, *p* = 0.09) [28]. 4) The Inspire II UAS device (Inspire Medical Systems, Inc., Maple Grove, MN, USA) is the only device currently approved by the US Food and Drug Administration (FDA). A sensor between the intercostal muscles detects respiratory effort from the chest, which is them analyzed by the IPG [29,30].

The Stimulation Therapy for Apnea Reduction (STAR) trial [31] examined the safety and efficacy of the Inspire Medical Systems device. The study included 126 CPAP-intolerant patients with moderate to severe OSA. Patients with body mass index (BMI) values greater than 32 kg/m^2^, AHI > 50 events per hour, central or positional sleep apnea, and/or concentric palatal collapse were not studied. During the 12-month extended follow-up period, the AHI and ODI both decreased (each *p* < 0.0001), falling from 29.3/h to 9.0 events/h and from 25.4 to 7.4/h, respectively. Secondary outcome measures showed improved quality of life as measured by the ESS and the Functional Outcomes of Sleep Questionnaire (FOSQ). Reductions in AHI (>50%) after 36 months and 48 months of follow-up, as well as improvements in subjective measures of sleepiness and quality of life, have also been reported [32,33].

There is mounting evidence that electrical stimulation of pharyngeal muscles is a promising, safe, and effective alternative to CPAP for the treatment of moderate to severe OSA. Hypoglossal nerve electrical stimulation significantly decreases AHI, ODI, and ESS values. The principal challenges regarding the implementation of UAS relate primarily to three issues. The first is that not all participants respond to this therapy, probably due to variable response to electrical activity and different mechanical displacement of the muscle in different patients. In the STAR trial, 43 of 126 (34%) participants did not respond. The second is the high cost. The third is the invasive nature of this treatment. These conditions limit accessibility in publicly funded healthcare systems [31,34]. Specific technical features will be needed to improve implantable devices effectively in the near future.

## 5. Pharmacological Treatment

Until a few months ago, very limited progress had been made in developing pharmacotherapies for OSA treatment, and there is currently no pharmacotherapy for OSA. The motor system controlling ventilation is complex, with neuronal activity reduced at sleep onset. The known reductions in upper airway muscle activity during sleep through serotonergic, noradrenergic, and cholinergic pathways have been investigated with the goal of improving pharmacological OSA treatment. These neurochemical mechanisms that are involved in sleep/wake-dependent control of respiration have led to the main hypothesis regarding pharmacological control for OSA.

Serotonin (5-hydroxytryptamine (5-HT)) has excitatory effects on hypoglossal motoneurons, and during sleep, there is a reduction in its delivery to upper airway dilator motor neurons [35,36]. At first, this decrease in endogenous serotonin was considered the main mechanism of upper airway collapsibility during sleep, secondary to the loss of GG activity in this state; this hypothesis is known as “the serotonin hypothesis.” Different works in animals and humans have tested the effect of serotonin on GG stimulation. Studies in rats demonstrated REM sleep–like GG atonia caused by loss of serotonergic inputs [36]. However, the acute effects of serotonin agents in humans increased GG activity during NREM sleep but did not improve OSA severity (measured by AHI) [37] or achieved limited success when combined with other drugs [38].

The central reduction in norepinephrine from wakefulness to sleep has been identified as one of the main causes of upper airway hypotonia. Recent research in animals has shown that noradrenergic processes can play a role in the mechanism of pharyngeal hypotonia that occurs during sleep [39]. Terazosin (an α1-adrenergic receptor antagonist) reduced GG activity during wakefulness and non-REM (NREM) sleep in rats, while phenylephrine (an α1 agonist) increased GG activity during this state, demonstrating that noradrenergic activity during NREM plays an important role in the hypotonia of pharyngeal muscles during sleep. Taranto-Montemurro et al. [40] tested the effect of desipramine (a tricyclic antidepressant with a relevant noradrenergic effect) in healthy adult subjects and found that it mitigated the decrease in tonic GG activity that occurs from wakefulness to NREM sleep (significantly increasing baseline tonic GG electromyography nearly to waking levels), reducing airway collapsibility but not altering GG phasic activity, muscle responsiveness to intrapharyngeal negative pressure, sleep architecture, or sleep efficiency. The same group also tested desipramine in OSA patients and found a reduction in pharyngeal collapsibility, but with limited effect on OSA severity as measured by AHI [41].

Nevertheless, REM atonia seems to be mediated by a muscarinic effect. Grace et al. [42] showed that muscarinic receptor antagonism in the hypoglossal motor pool prevents the inhibition of GG activity throughout REM sleep without pronounced effects during wakefulness or NREM in rats, demonstrating that GG muscle tone in this phase is regulated by muscarinic receptors.

**Figure 8:**
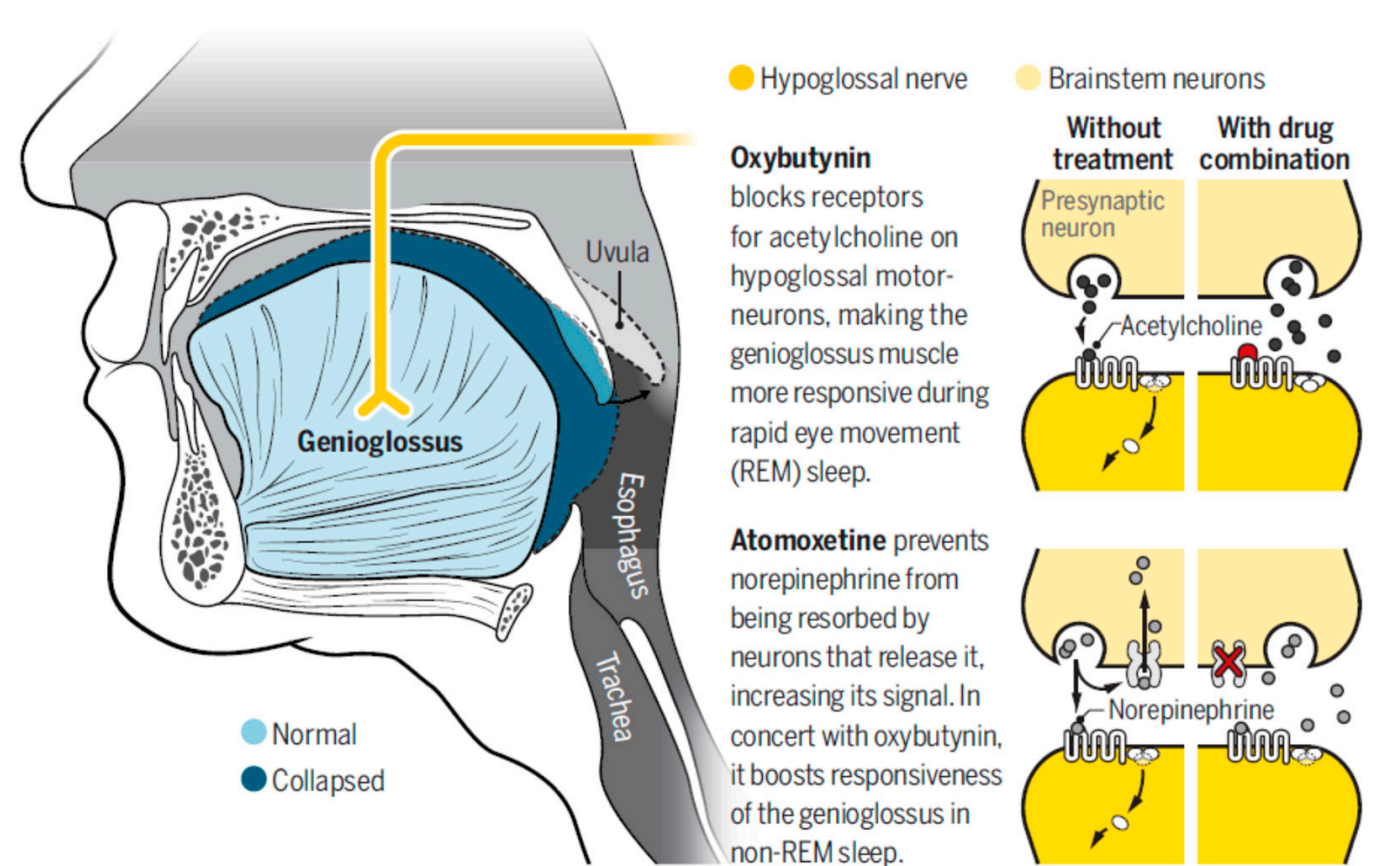
Effect of atomoxetine and oxybutynin (ato-oxy) on hypoglossal nerve and genioglossus muscle responsiveness. With permission from Wadman M. Drug pair shows promise for treating sleep apnea. Reprinted with permission from AAAS [44].

Based on this knowledge, a combination therapy (a norepinephrine reuptake inhibitor and a muscarinic blocker) designed to optimally modulate GG muscle tone across NREM and REM sleep was tested [42] (Figure 8). For the first time, Taranto-Montemurro et al. [43] showed an AHI decrease (28.5 to 7.5 events/h; median change: 63%; *p* < 0.001) in a one-night randomized placebo-controlled double blind crossover trial in 20 patients and an increase in the oxygen saturation nadir in patients with mild to moderate OSA treated with ato-oxy, a combination of atomoxetine (a norepinephrine reuptake inhibitor) and oxybutynin (an antimuscarinic agent). The effect seems to be directly related to the effect on the GG muscle, and the authors found greater responsiveness to ato-oxy than to placebo in GG electromyography measurements. When the drugs were tested separately (nine patients), there were no significant effects on AHI. This approach has been the first effective OSA pharmacotherapy, and it has been a major step for OSA pharmacological treatment, but additional efforts should be made to demonstrate the sustained effect of the drugs over time and to identify the clinical response, doses, side effects, and groups of patients in whom the drug can be effective. Important considerations about this combination of drugs should be taken into account: (1) the effect has been tested only in a one-night trial. The sustained effect of the drugs should be demonstrated in longer trials to confirm the efficacy of the treatment in the long term. (2) Since atomoxetine can produce an increase in blood pressure and heart rate, it is contraindicated in severe cardiovascular conditions (which are common in OSA patients). (3) The combination did not reduce arousals and increased N2; furthermore, REM sleep suppression has been known to occur in relation to oxybutynin. Sleep architecture should be studied with different doses and longer treatments, and the effects of the treatment on somnolence and tiredness should also be examined. (4) The clinical response has not been evaluated. Event suppression is important in OSA control, but symptom control should also be demonstrated. (5) The effect was studied in only 20 patients; the response needs to be determined in a larger population. (6) CPAP-treated patients stopped treatment only during the study night. It is likely that a longer washout period would be more effective in determining the real AHI (five out of 20 participants exhibited an AHI<10 events/h on placebo). (7) A group of patients (8 of the 15 with AHI>10 events/h) still had a residual AHI≥10 events/h after treatment, suggesting that the effectiveness of the potential therapy could be specific to a subgroup of patients and that testing is needed in different OSA phenotypes (subgroups of resistant OSA patients).

Obviously, these findings open exciting new possibilities for OSA treatment, probably for patients with a specific phenotype and/or in combination with other treatments, but it would be premature to use this combination as a treatment option for OSA.

## 6. Myofunctional Therapy (MT)

The pathophysiological causes of OSA in adults include an anatomically compromised upper airway (narrow pharynx or increased upper airway length), inadequate responsiveness of the pharyngeal dilator muscles during sleep, a low respiratory arousal threshold, and a high loop gain [45]. However, the pathophysiology of OSA in childhood is complex and poorly understood. Currently, the most commonly hypothesized main cause of pediatric OSA is an anatomically (adenotonsillar hypertrophy) or functionally narrowed upper airway [46].

As mentioned, upper airway dilator muscles can contribute to the genesis of OSA because they are crucial to the maintenance of pharyngeal patency. As previously demonstrated, poor GG muscle responsiveness to negative pharyngeal pressure and changes in the activity of oropharyngeal muscles during sleep play very important roles in maintaining an open airway during sleep. For this reason, recent studies have explored the effects of oropharyngeal exercises and other airway activity (singing; playing the didgeridoo or other instruments) that focus oral cavity and oropharyngeal structures as a complementary technique for treating OSA [47,48]. Myofunctional therapy (MT) or oropharyngeal exercises that lead to changes in dysfunctional upper airway muscles have been suggested to be effective for reducing OSA severity in adults along with associated symptoms, especially when the severity of the disease is moderate [49].

Since 1918, MT has been described to increase mandibular growth and to improve nasal breathing and facial appearance [50]; subsequently, in the 1990s, Guimarães et al. [51] proposed MT as a new tool in the management of OSA. However, as the use of full-night polysomnography (PSG) for the diagnosis of OSA was not widespread by that time, only the clinical situation and the symptoms of the patients were initially considered to apply this therapy. In recent years, partly due to the dissemination of knowledge and partly due to the increasing availability of resources, OSA has attached the attention of many different specialists, and there have been a growing number of studies exploring the effect of MT in OSA [49,52,53,54,55]. MT is a treatment method for subjects with orofacial myofunctional disturbance that may interfere with orofacial development or function. This approach is built on isotonic and isometric exercises that promote the sensitivity, proprioception, mobility, coordination, and strength of orofacial structures [49,52]. MT also promotes the appropriate efficiency of respiration and other functions such as mastication, swallowing and speech. Oropharyngeal exercises are derived from speech language pathology and include different types of soft palate, tongue, and facial muscle exercises as well as stomatognathic function exercises. The most extensive MT exercises were described by Guimarães et al. [49], and this protocol was applied in other studies in its original form or with some modifications [56].

In randomized studies evaluating the effect of MT on sleep-disordered breathing based on full PSG data, results have shown a significant reduction in AHI in adults with moderate OSA [49,56,57,58], along with a reduction in AI [49] and an increased minimum percentage of oxygen saturation [49]. One recent systematic review and meta-analysis included a total of nine studies performed in adults (120 patients, age 44.5 ± 11.6 years) that reported PSG and/or sleepiness outcomes and evaluated the impact of MT on OSA [59]. The majority of the adult patients had moderate OSA. The pre- and post-MT AHI decreased from 24.5 ± 14.3/h to 12.3 ± 11.8/h, with a mean difference of −14.26 (95% CI −20.8–7.54, *p* > 0.0001), which was a 50% reduction. The lowest oxygen saturation improved in 82 patients from 83.9 ± 6.0% to 86.6 ± 7.3%. Only two studies (including 43 patients) were randomized controlled trials (RCTs) [49,55]. The nonrandomized studies reported that after MT, patients with mild or moderate OSA showed significant reductions in AHI [60,61,62] and AI [60,61,62] and an increase in lowest oxygen saturation [60,61,62]. Only one study analyzed the effects of MT in a group of patients with severe OSA; this study showed a reduction in mean AHI, but the reduction was nonsignificant [62].

With regard to snoring, Ieto et al. [58] performed an RCT in 39 patients to evaluate the effects of oropharyngeal exercises in mildly symptomatic patients with a primary complaint of snoring and a diagnosis of primary snoring or mild to moderate OSA. The results showed a subjective improvement in snoring intensity reported by a bed partner or perceived by the patient as well as significant decreases in the snore index (total number of snores/total sleep time) from 99.5 to 48.2, *p* = 0.041, and in the total snore index (sound intensity power/total sleep time) from 60.4 to 31.0, *p* = 0.033. Other authors also showed a 72.4% reduction in snoring after MT (14.05 ± 4.89% to 3.87 ± 4.12% before and after MT, respectively, *p* < 0.001) [60].

Subjective sleepiness, assessed by the ESS, also improves after MT. Camacho et al. [59] showed a significant improvement in sleepiness after MT, with a reduction from 14.8 ± 3.5 to 8.2 ± 4.1 in the ESS in 75 patients. In a systematic review [63], patients with a mean baseline ESS score ranging from 12 ± 2.6 to 15.4 ± 2.3 revealed improved scores after MT, with a mean reduction of six points [49,56,57,60,62]. However, in a group of patients who were not sleepy [56], with a median ESS score of 7.0 (3–11), and in a group of patients with severe OSA and an ESS score of 20.9 ± 6.2 [62], no significant changes were identified.

The effect of MT on quality of life has also been studied, and quality of life seems to improve after treatment with MT alone or in association with CPAP [56]. In two studies that investigated morning headache symptoms [60,61], only one detected a reduction (from 60% to 20%) in the number of patients with this complaint [60].

In children, a prospective RCT was conducted in which postadenotonsillectomy patients were randomized to either oropharyngeal exercises or a control group [64]; the investigators found that after two months of MT, the AHI reduced from 4.87 ± 3.0/h to 1.84 ± 3.2/h (*p* = 0.004, a 62% reduction) in the treatment group. The control group had minimal changes in AHI during the 2-month interval. In a retrospective review, Guilleminault et al. [52] evaluated 24 children who were treated with a combination of adenotonsillectomy and palatal expansion (AHI 0.4 ± 0.3/h). Eleven of the children received MT (intervention group), and 13 children did not receive MT. They were followed up for four years; after this period, the children who received MT remained cured of OSA (AHI 0.5 ± 0.4/h), whereas the children who were never trained to perform the exercises subsequently experienced OSA recurrence (AHI 5.3 ± 1.5/h).

In summary, MT is effective for the treatment of OSA in adults, reducing AHI, AI, and snoring, as well as improving subjective symptoms related to daytime sleepiness, sleep, and quality of life, mainly in mild to moderate OSA. However, long-term studies are needed. In children with residual apnea after adenotonsillectomy, MT sustainably decreases the AHI.

## 7. Clinical Applicability

OSA is a heterogeneous and multicomponent syndrome that requires a detailed evaluation (clinical presentation, comorbidities, physical exploration, sleep test, etc.) to make a therapeutic decision. There is no ideal treatment for all OSA patients, and monotherapy is an error management strategy for most patients. Therapies that target one or more of these causes can lead to a new approach in OSA treatment. Intensive behavioral lifestyle interventions in diet, exercise, sleep hygiene, avoiding alcohol and sedatives, and sleeping in a supine position are mandatory in every patient. The reversible causes of OSA should always be explored, and these issues, if present, should be the target of first-line therapy. When a definitive treatment is not possible, different therapeutic options should be considered, and a combination of treatment could be necessary. For this aim, a better characterization of the mechanism involved in OSA, upper airway narrowing due to anatomical factors and non-anatomical phenotypes is needed for a customized treatment.

New therapeutic options centered on GG function could play a role in this treatment strategy in isolation in specific groups of patients or in combination with other therapies. Classical treatments such as CPAP and OAs have failed to demonstrate universal effectiveness in all types of patients.

Hypoglossal nerve stimulation has achieved sustained and significant improvement in AHI, quality of life and sleepiness, but its success depends on individual characteristics, and some subjects are nonresponders. This approach could be an additional effective tool in well-selected patients with moderate to severe OSA; however, it is invasive and costly. Moreover, hypoglossal nerve stimulation could be useful in patients with severe sleep apnea who do not tolerate CPAP, but the placement of the stimulator requires experience. Additionally, this treatment modality is not useful in patients with morbid obesity, and candidates should be evaluated carefully.

Pharmacological treatment is a new area of research in which initial positive results have been published. The drug combination ato-oxy has been demonstrated to significantly decrease AHI and improve oxygen saturation during sleep in OSA patients. This combination would probably be a good option in certain groups of patients in the future, but larger and longer studies are needed. At present, it would be premature to use pharmacological agents to increase muscle activity for OSA treatment.

MT has demonstrably improved AHI, nadir saturation and subjective sleepiness. This approach could be a useful tool in nonobese patients with mild to moderate OSA or could improve the effectiveness or adherence of CPAP treatment by reducing the absolute pressure required. One of the most important limitations is that MT requires high patient adherence to the therapy because exercises must be performed by the patient at least two to three times per day. This condition limits the clinical applicability of MT.

OSA management must be personalized and avoid a one-size-fits-all approach. In evaluating the pathophysiological causes, it is mandatory to apply potential novel and/or combined therapeutic options that could be used independently or in combination with classical OSA therapies.

**Figure 9:**
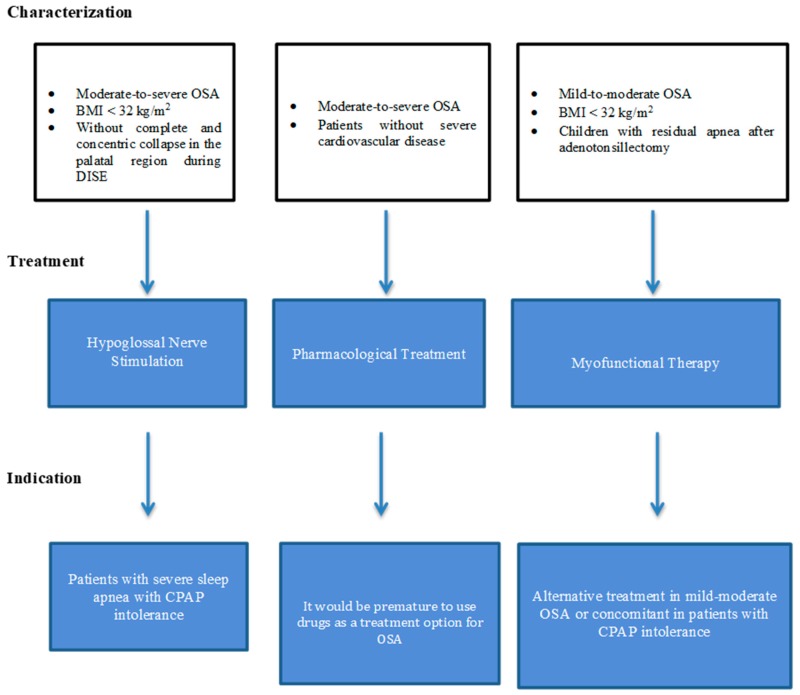
Alternatives to continuous positive airway pressure (CPAP) treatment: a diagram suggesting a phenotype-based treatment for adult obstructive sleep apnea patients and possible indications. OSA: obstructive sleep apnea; CPAP: continuous positive airway pressure; BMI: body mass index.

Table 1 summarizes the main results and conclusions from OSA-GG treatment, and Figure 9 summarizes the patient phenotype that can benefit from each treatment and possible indications.

## 8. Conclusions

OSA is a heterogeneous condition with multifactorial pathophysiology. New therapeutic strategies need to be added to classical OSA management for complete control of the disease. OSA treatments focusing on GG muscle in isolation may be problematic, but these therapies can be useful in specific groups of patients or in combination with another treatment.

Nerve electrical stimulation is a safe and effective treatment for moderate to severe OSA patients and could be an alternative to CPAP. The main problems with this treatment are its cost and its invasive nature.

Exciting new possibilities for OSA treatment have emerged with the recent results of pharmacological treatment for OSA, but it would be premature to use ato-oxy as a treatment option for OSA at present. In the future, it could be useful for a specific patient phenotype and/or in combination with other treatments.

MT could be a useful tool in nonobese patients with mild to moderate OSA. It can improve the effectiveness or patient adherence of CPAP treatment by reducing the absolute pressure, but it requires high patient adherence to the exercise regimen itself.

## Figures and Tables

**Table 1:** Summary of the main results in OSA-GG treatment and conclusions.

Reference	Severity	*n*	Treatment	Follow-Up	Main Effect	Conclusion
**Hypoglossal Nerve Electrical Stimulation**						
Strollo, 2014 [31]	Moderate to severe OSA	126	Inspire II Upper Airway Stimulation	12 months	Decreases AHI 68% (from 29.3 to 9.0 events/h). Decreases ODI score 70% (from 25.4 to 7 events/h). Improve EDS and quality of life.	(1) Safe and effective for the treatment of moderate to severe OSA. (2) Could be an alternative to CPAP. (3) Significant improvements in objective (AHI) and subjective measurements. (4) Its invasive nature limits its application.
Woodson, 2016 [32]	Moderate to severe OSA	116	Inspire II Upper Airway Stimulation	36 months	Decreases AHI > 50% (from 28.2 to 6.2 events/h). Improves quality of life.	
Gillespie, 2017 [33]	Moderate to severe OSA	91	Inspire II Upper Airway Stimulation	48 months	Improves ESS and quality of life.	
**Pharmacological Treatment**						
Berri, 1999 [37]	Severe OSA	8	Paroxetine	Single dose	Increases peak inspiratory GG activity during NREM. Does not improve AHI.	(1) Exciting new possibilities for OSA treatment. (2) Probably suitable for a determined phenotype of patients and/or in combination with another treatments. (3) It would be premature to use this combination as a treatment option for OSA at present.
Prasad, 2010 [38]	AHI > 10	35	Ondansetron + fluoxetine	Days 7, 14 and 28	Decreases AHI 40% at high dose (12.9 events/h reduction in AHI). Does not improve EDS.	
Taranto-Montemurro, 2016 [41]	AHI > 15	14	Desipramine	Single dose	Decreases pharyngeal collapsibility (Pcrit). Very little effect on AHI.	
Taranto-Montemurro, 2019 [43]	15/20 patients with OSA on placebo (AHI>10 events/h)	20	Atomoxetine + oxybutynin	Single dose	Median AHI change of 63% (from 28.5 to 7.5 events/h). Increases nadir oxygen saturation. Increases GG responsiveness.	
**Myofunctional Therapy**						
Guimarães, 2009 [49]	Moderate OSA	31	Upper airway exercises	3 months	Decreases AHI from 22.4 to 13.7/h. Increases nadir oxygen saturation. Improves EDS.	(1) It could be a useful tool in nonobese patients with mild to moderate OSA. (2) Can improve the effectiveness or patient adherence of CPAP treatment by reducing the absolute pressure required. (3) One of the most important limitation is that it requires high patient adherence to the therapy.
Diaferia, 2013 [55]	Moderate to severe OSA	100	Speech therapy	3 months	Improves quality of life.	

OSA: obstructive sleep apnea; AHI: apnea-hypopnea index; ODI: oxygen desaturation index; EDS: excessive daytime sleepiness; CPAP: continuous positive airway pressure; ESS: Epworth Sleepiness Scale; GG: genioglossus.
